# ARC is essential for maintaining pancreatic islet structure and β-cell viability during type 2 diabetes

**DOI:** 10.1038/s41598-017-07107-w

**Published:** 2017-08-01

**Authors:** Wendy M. McKimpson, Min Zheng, Streamson C. Chua, Jeffrey E. Pessin, Richard N. Kitsis

**Affiliations:** 1 Department of Medicine, Albert Einstein College of Medicine, Bronx, NY 10461 USA; 2 Department of Cell Biology, Albert Einstein College of Medicine, Bronx, NY 10461 USA; 3 Department of Neuroscience, Albert Einstein College of Medicine, Bronx, NY 10461 USA; 4 Department of Molecular Pharmacology, Albert Einstein College of Medicine, Bronx, NY 10461 USA; 5 Wilf Family Cardiovascular Research Center, Albert Einstein College of Medicine, Bronx, NY 10461 USA; 6 Einstein-Mount Sinai Diabetes Research Center, Bronx, NY 10461 USA; 7 Albert Einstein Cancer Center, Albert Einstein College of Medicine, Bronx, NY 10461 USA; 8 Present Address: Department of Medicine (Endocrinology), Columbia University Medical Center, New York, NY 10032 USA

## Abstract

Pancreatic β-cell loss through apoptosis is an important disease mechanism in type 2 diabetes. Apoptosis Repressor with CARD (ARC) is a cell death inhibitor that antagonizes multiple death programs. We previously reported that ARC is abundant in pancreatic β-cells and modulates survival of these cells *in vitro*. Herein we assessed the importance of endogenous ARC in maintaining islet structure and function *in vivo*. While generalized loss of ARC did not result in detectable abnormalities, its absence in *ob/ob* mice, a model of type 2 diabetes, induced a striking pancreatic phenotype: marked β-cell death, loss of β-cell mass, derangements of islet architecture, and impaired glucose-stimulated insulin secretion *in vivo*. These abnormalities contributed to worsening of hyperglycemia and glucose-intolerance in these mice. Mechanistically, the absence of ARC increased levels of C/EBP homologous protein (CHOP) in wild type isolated islets stimulated with ER stress and in *ob/ob* isolated islets at baseline. Deletion of CHOP in *ob/ob*; ARC −/− mice led to reversal of β-cell death and abnormalities in islet architecture. These data indicate that suppression of CHOP by endogenous levels of ARC is critical for β-cell viability and maintenance of normal islet structure in this model of type 2 diabetes.

## Introduction

Hyperglycemia during type 2 diabetes is initially mediated by insulin resistance, but later inadequate production of insulin contributes significantly to progression of disease. This failure of β-cells may result from dysfunctional insulin secretion, cellular dedifferentiation, and cell death^1^. The molecular mechanisms that mediate β-cell death during diabetes, however, remain incompletely understood.

While multiple death programs may operate in cells, β-cell death during type 2 diabetes appears to occur primarily by apoptosis. Apoptosis is mediated by two central pathways, one utilizing cell surface death receptors and the other involving the mitochondria and endoplasmic reticulum (ER)^2^. Both pathways lead to the activation of a hierarchy of caspases, a subclass of cysteine proteases^3^. In the death receptor pathway, soluble and membrane-bound extracellular ligands bind cell surface receptors to trigger the assembly of the Death Inducing Signaling Complex (DISC), a multi-protein complex in which caspase-8 is activated^4^. In contrast, the mitochondrial-ER pathway transduces a broader array of death stimuli including metabolic, oxidative, and ER stressors as well as loss of soluble survival factors and support from extracellular matrix. These stimuli act through Bcl-2 family proteins Bax and Bak to trigger the release of cytochrome c (and other apoptogens) from mitochondria. Cytosolic cytochrome c induces the formation of the apoptosome, a multiprotein complex in which caspase-9 is activated^5^. Caspases-8 and -9 then cleave and activate effector caspases-3, -6, and -7, which cut multiple cellular substrates to bring about apoptotic cell death.

Death stressors of importance in type 2 diabetes include excesses in extracellular glucose and lipids. Although not mechanistically well understood, these stimuli induce ER stress, which is thought to be an important driver of β-cell apoptosis^6^. Genetic manipulations in the mouse suggest that both death receptor and mitochondrial-ER apoptosis pathways are involved in β-cell death in the pathogenesis of type 2 diabetes. For example, β-cell-specific deletion of caspase-8 in the death receptor pathway^7^ or overexpression of Bcl-2^8^, an inhibitor of mitochondrial apoptogen release, both protect against diabetes. However, the alterations in death signaling that actually mediate the natural pathogenesis of this syndrome are poorly understood.

Cell death pathways are under negative as well as positive control. ARC (Apoptosis Repressor with CARD (Caspase Recruitment Domain)) is an endogenous inhibitor of cell death that was initially thought to be restricted to cardiac and skeletal myocytes and some neuronal populations^9, 10^. More recent work, however, has shown that ARC expression is induced in multiple cancers^11–13^, and that it is abundant at baseline in human and mouse β-cells *in vivo* ^14^. While most cell death inhibitors target a single pathway, ARC antagonizes both central death pathways through protein-protein interactions involving its CARD^15^. Inhibition of the death receptor pathway is mediated through direct interactions of ARC with the death receptor Fas and with its adaptor protein FADD, which preclude assembly of the DISC. ARC suppresses the mitochondrial-ER pathway by direct binding to Bax, which blocks Bax conformational activation.

We previously employed gain and loss of function approaches using *in vitro* systems to define an inhibitory role for ARC in β-cell death elicited by ER stressors and lipotoxicity^14^. Given the complexity of diabetes and the possibility of redundancy among multiple cell death inhibitors, however, we wished to assess the role of ARC *in vivo*. Using a series genetic mouse models, we now report that ARC is critical not only for β-cell survival during type 2 diabetes but also for maintenance of islet structure *in vivo*. Moreover, ARC-mediated suppression of the ER stress mediator C/EBP homologous protein (CHOP) underlies these effects.

## Results

### Deletion of ARC does not result in baseline abnormalities in pancreatic structure or hyperglycemia

**Figure 1:**
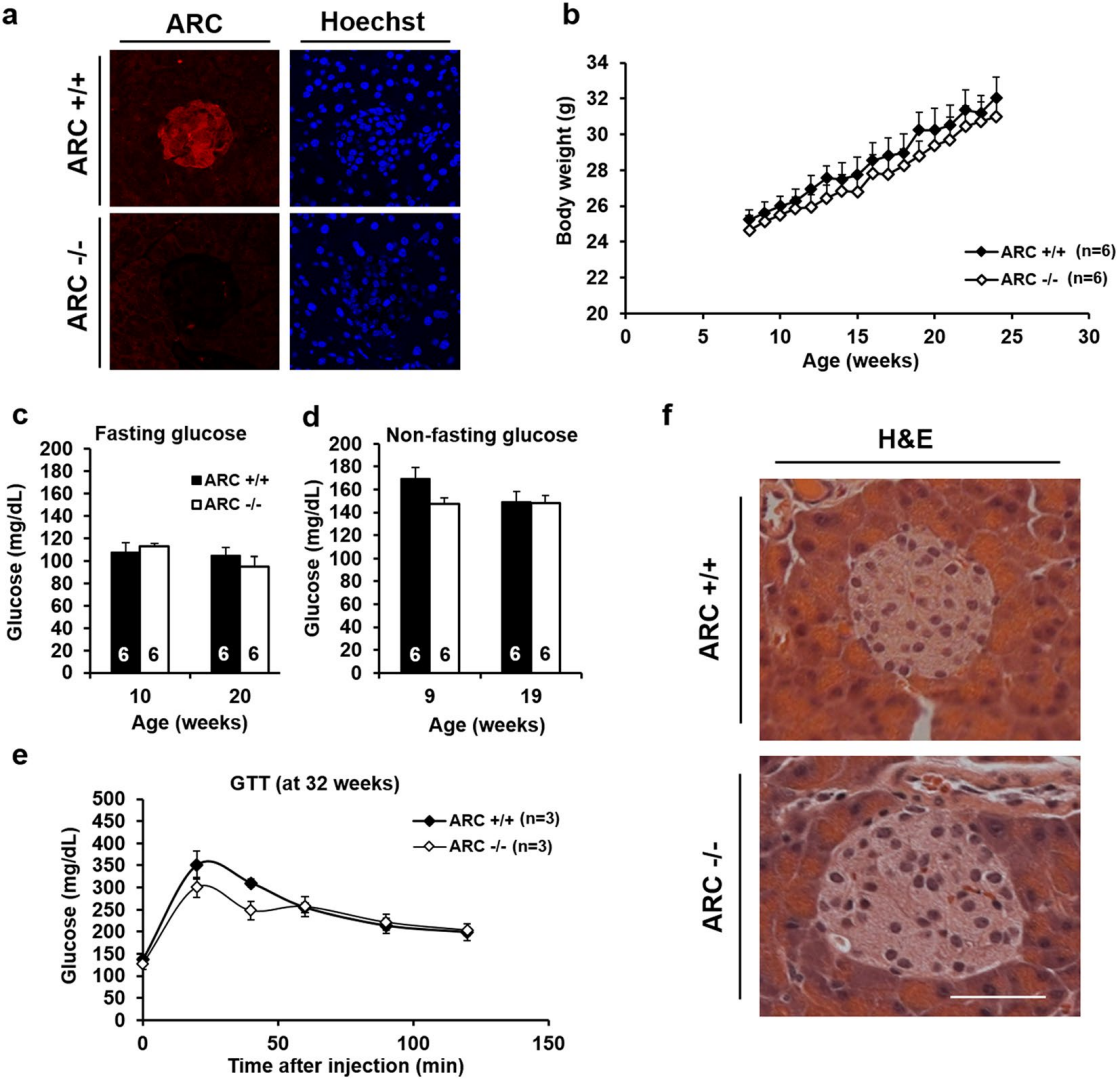
ARC −/− mice do not manifest abnormalities in glucose homeostasis and pancreatic structure. (**a**) Immunofluorescence of mouse pancreatic tissue for ARC (red). (**b**) Body weights. (**c**) Fasting blood glucose concentrations. (**d**) Non-fasting blood glucose concentrations. (**e**) Glucose tolerance test (GTT). (**f**) Hematoxylin and eosin (H&E) staining of pancreatic tissue. Scale bar 50 μM. No statistically significant differences were observed. This experiment was repeated in a second independent cohort of mice with similar results.

We had previously generated mice with germ line deletion of the entire open reading frame for ARC encoded by the *Nol3* gene^16, 17^ and, for clarity, will refer to homozygous knockouts as ARC −/−. Immunostaining confirmed the absence of ARC protein in pancreatic islets of knockout mice (Fig. 1a), as we have shown previously for other tissues in which ARC is normally expressed^16, 17^. Wild type and ARC −/− mice were indistinguishable with respect to body weights, fasting and non-fasting blood glucose concentrations, glucose tolerance, and pancreatic histology (Fig. 1b–f). These data indicate that ARC is not required for normal pancreatic structure or glucose homeostasis under basal conditions.

### ARC is essential for β-cell viability and islet structure in a diabetic context

**Figure 2:**
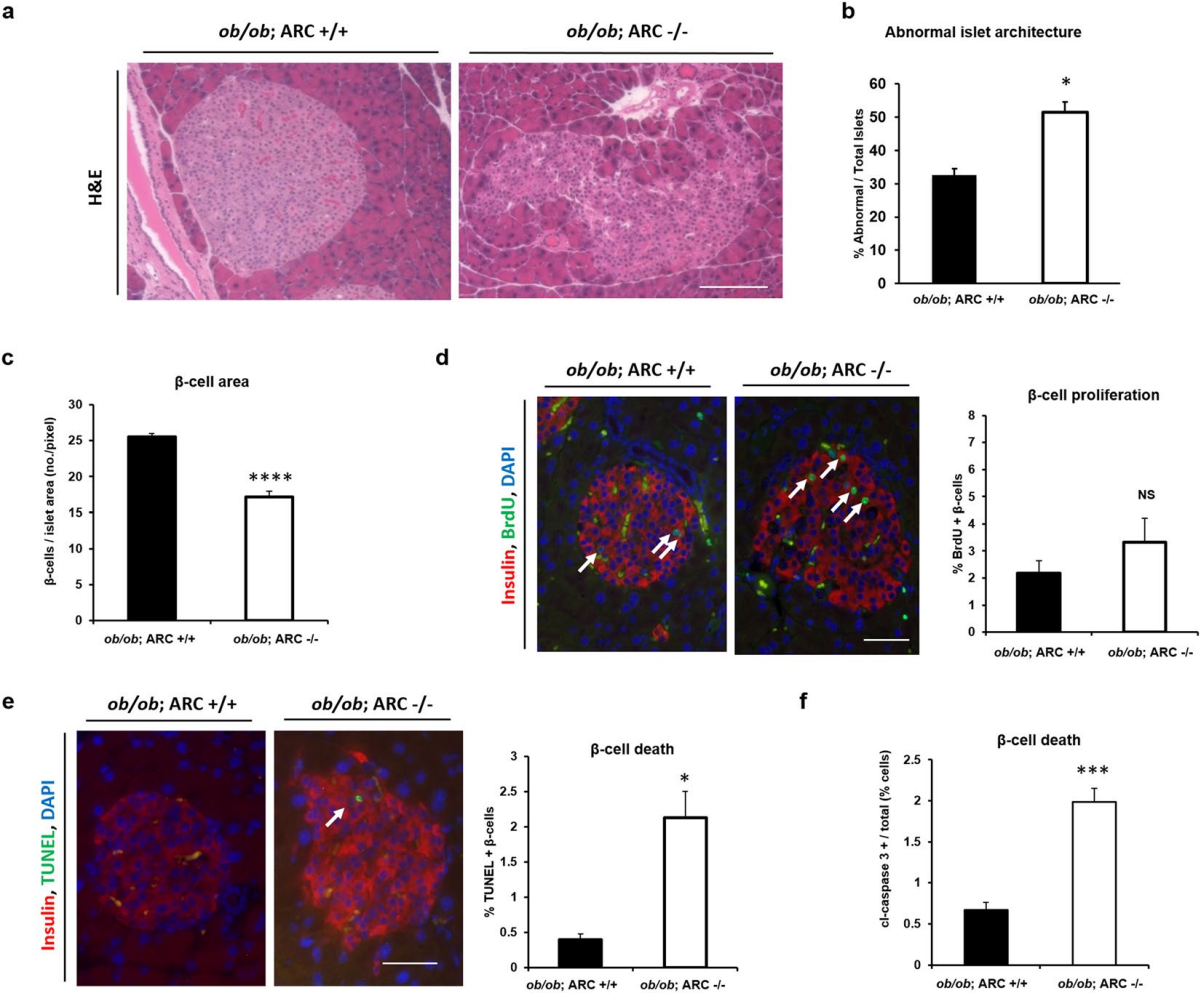
Deletion of ARC results in β-cell death and abnormalities in islet architecture in *ob/ob* mice. (**a**) Hematoxylin and eosin (H&E) staining of pancreatic tissue from 24 week old mice. Scale bar 100 μM. (**b**) Percentage of abnormal islets as determined from H&E staining. (**c**) β-cell area. (**d**) β-cell proliferation as determined by incorporation of BrdU into nuclei of insulin-positive cells. Arrows demarcate BrdU-positive β-cells. Scale bar 50 μM. (**e**) and (**f**) β-cell apoptosis as determined by TUNEL (panel e) and percentage of β-cells with cleaved (cl) caspase-3 (panel f). In panel e, arrow indicates TUNEL-positive β-cell, and scale bar 50 μM. NS is not significant. *P < 0.05, ***P < 0.001, and ****P < 0.0001.

To assess the importance of ARC in β-cell survival under the stressed conditions of type 2 diabetes, we crossed ARC −/− mice with *ob/ob* mice, a leptin deficient line that exhibits obesity and type 2 diabetes. The absence of ARC on the *ob/ob* background resulted in marked disorganization in islet architecture compared to control animals (Fig. 2a). Whereas *ob/ob*; ARC +/+ mice had a ratio of 2:1 normal to abnormal islets, *ob/ob*; ARC −/− had equal numbers of abnormal and normal islets (Fig. 2b). Diabetic mice lacking ARC also had a significant decrease in β-cell mass (Fig. 2c), a difference not explained by changes in the rates of β-cell proliferation (Fig. 2d). In contrast, there was a 4-fold increase in rates of apoptosis as assessed by TUNEL (Fig. 2e) and immunostaining for cleaved (active) caspase-3 (Fig. 2f). These data indicate that, in the *ob/ob* context, ARC is essential for β-cell survival and maintenance of normal islet structure.

**Figure 3:**
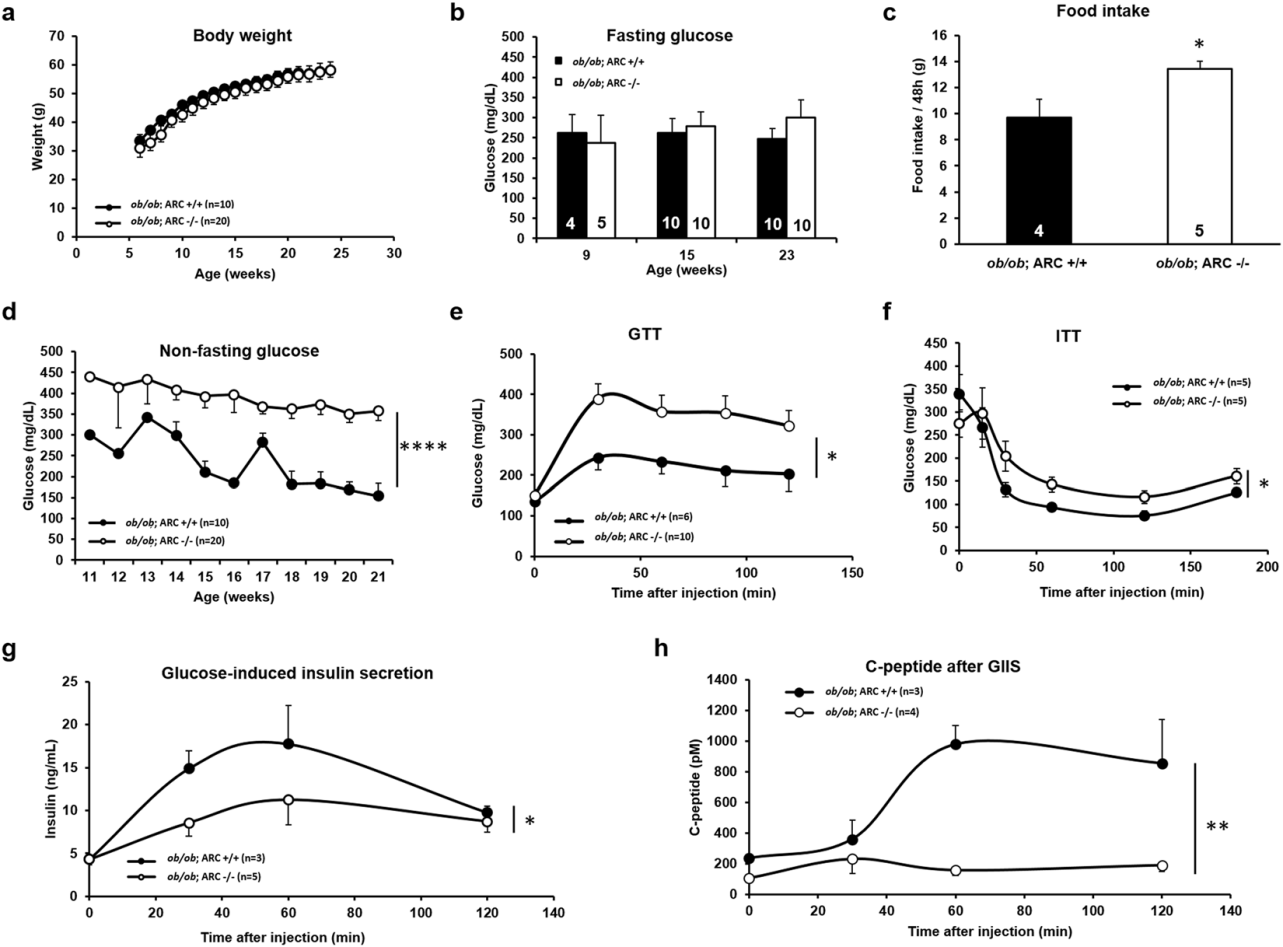
*ob/ob;* ARC −/− mice exhibit hyperglycemia, glucose intolerance, β-cell dysfunction, and hyperphagia. (**a**) Body weights. (**b**) Fasting blood glucose concentrations. (**c**) Food intake over 48 h. (**d**) Non-fasting blood glucose concentrations. (**e**) Glucose tolerance test (GTT). (**f**) Insulin tolerance test (ITT). (**g**) Glucose-induced insulin secretion (GIIS). (**h**) Plasma C-peptide during GIIS. *P < 0.05. **P < 0.01. ****P < 0.0001. Area under the curve measurement was performed on the first three time points for panel g. Unless otherwise indicated, experiments were performed in mice 20–28 weeks of age. These experiments were repeated in a second independent cohort of mice with similar results.

We also characterized the metabolic phenotype of *ob/ob*; ARC −/− mice. Loss of ARC did not affect body weight or fasting plasma glucose concentrations, but non-fasting blood glucose levels were markedly and persistently elevated starting at 11 weeks of age as was food intake (Fig. 3a–d). Deletion of ARC also impaired glucose tolerance (Fig. 3e). While both *ob/ob*; ARC +/+ and *ob/ob*; ARC −/− mice exhibited severe insulin resistance as demonstrated by the need to employ a high dose of insulin (5 U/kg) to decrease blood glucose concentrations, deletion of ARC exacerbated this abnormality to only a minor degree (Fig. 3f). Importantly, consistent with the loss of β-cell mass in *ob/ob*; ARC −/− mice (Fig. 2c), glucose-stimulated secretion of insulin and C-reactive peptide was impaired compared with *ob/ob*; ARC +/+ mice (Fig. 3g and h).

### ARC maintains β-cell viability and islet architecture through suppression of CHOP induction

**Figure 4:**
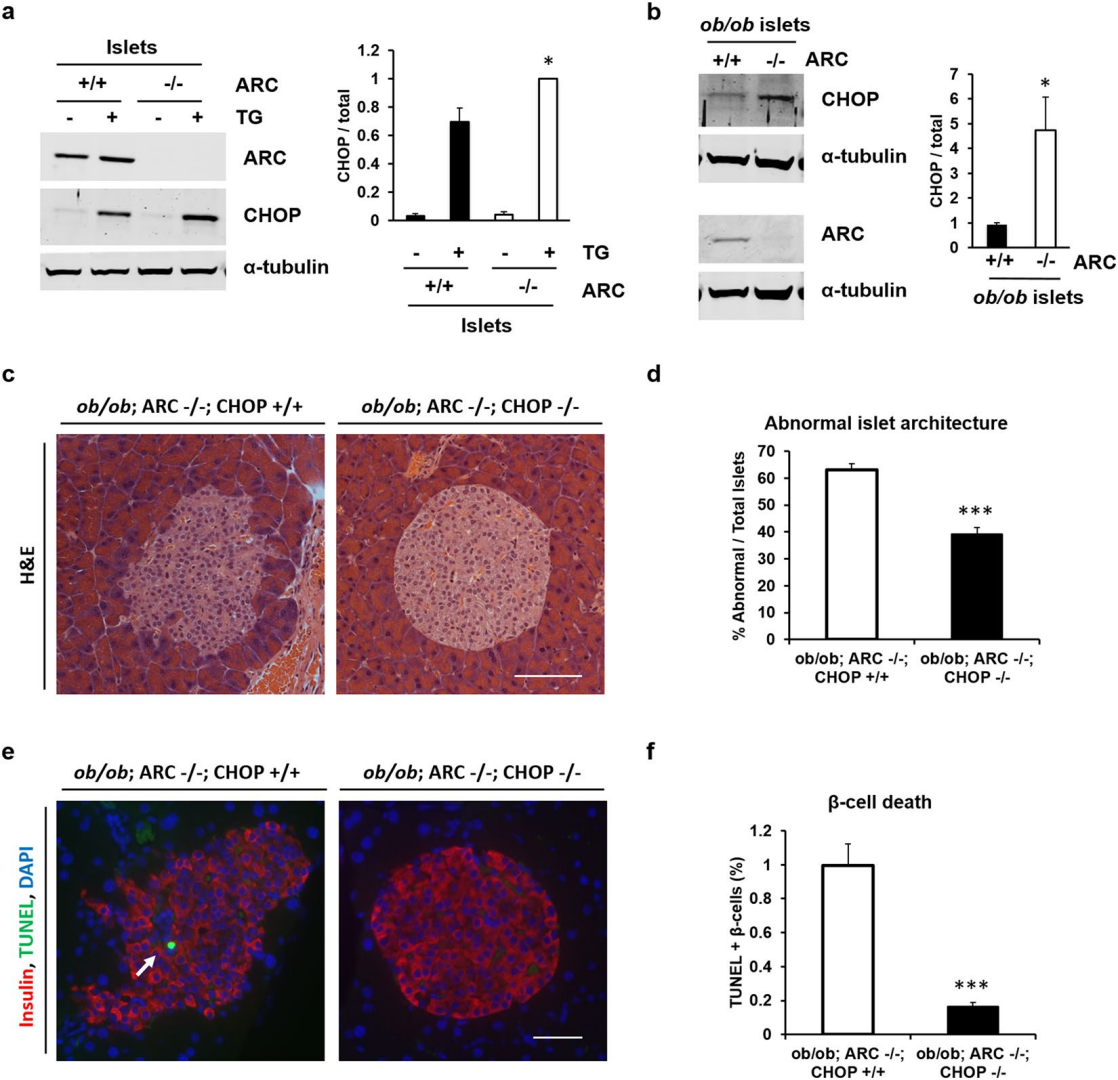
Deletion of CHOP restores islet architecture and β-cell viability in *ob/ob*; ARC −/− mice. (**a**) Immunoblot (left) and quantification (right) of islets isolated from indicated mice and treated overnight with ER stressor thapsigargin (TG, 1 μM). Graph is average of three independent experiments. (**b**) Immunoblot (left) and quantification (right) of islets isolated from *ob/ob*; ARC +/+ and *ob/ob*; ARC −/− mice. Graph is average of three samples for each genotype. (**c**) Pancreatic tissue from 24 wk old mouse stained with Hematoxylin and eosin (H&E). (**d**) Percentage of abnormal islets as determined from H&E staining. (**e**) β-cell apoptosis as determined by TUNEL (green) and insulin (red) staining. Arrow indicates TUNEL-positive β-cell. (**f**) Quantification of TUNEL staining. Scale bar 100 μM. ***P < 0.001.

ER stress is a major component in the pathogenesis of type 2 diabetes^18, 19^, and induction of the ER stress mediator CHOP has been implicated in β-cell death^20, 21^. We previously demonstrated in cultured β-cells that overexpression of ARC blunts the accumulation of CHOP in response to ER stressors^14^. To assess whether endogenous levels of ARC suppress the accumulation of CHOP, we treated islets isolated from ARC +/+ and ARC −/− animals not carrying the *ob/ob* mutation with thapsigargin to induce ER stress. Loss of ARC resulted in increased induction of CHOP (Fig. 4a). Moreover, islets isolated from *ob/ob;* ARC −/− mice exhibited marked elevations in CHOP at baseline compared with islets isolated from *ob/ob*; ARC +/+ mice (Fig. 4b). These data demonstrate that endogenous ARC suppresses CHOP levels. To determine whether the observed derangements in islet tissue architecture and β-cell death resulting from ARC absence are mediated by CHOP, we generated *ob/ob*; ARC −/−; CHOP −/− mice. The absence of CHOP rescued both the islet disorganization and β-cell death (Fig. 4c–f). We conclude that endogenous levels of ARC maintain β-cell survival and islet structure in *ob/ob* mice through suppression of CHOP.

## Discussion

While ARC knockdown renders cultured β-cells and isolated islets more susceptible to stress-induced death *in vitro* ^14^, loss of function phenotypes *in vitro* sometimes do not persist in the more complex *in vivo* environment because of redundancy from functionally similar molecules. Despite the expression of other cell death inhibitors *in vivo* ^8, 22^, we found that ARC is essential for β-cell survival in islets of *ob/ob* mice, although it is dispensable under non-stressed conditions. Further, the absence of ARC in *ob/ob* mice resulted in unexpected derangement of islet architecture. While our data show that both β-cell apoptosis and changes in islet architecture are CHOP-dependent, the mechanistic relationship of these two processes remains unclear. Although they may occur in parallel, it is intriguing to consider that one may be upstream of the other. For example, cell death-related alterations in β-cell structure or secretion might remodel surrounding tissue organization and/or, conversely, disruption of tissue organization could deprive β-cells of critical survival signals.

While loss of β-cell mass and accompanying decreases in glucose-stimulated insulin secretion contribute to exacerbation of hyperglycemia and the impaired ability to handle a glucose load in *ob/ob*; ARC −/− mice, the generalized absence of ARC in this mouse model leaves open the possibility of contributions from other tissues. For example, although only mildly dampened, decreases in insulin sensitivity in *ob/ob*; ARC −/− mice may be one such factor and could reflect loss of ARC in skeletal muscle and fat - but likely not liver where ARC expression is not detected even in wild type mice^10, 23^. In addition, hyperphagia may contribute to the fed hyperglycemia and be related to loss of ARC in neuronal populations that are known to be involved with appetite^24^. The generation of mice with a floxed *Nol3* allele will be necessary to dissect contributions to the hyperglycemia from various tissues.

ARC overexpression blunts the induction of CHOP in cultured β-cells challenged with experimental and physiological ER stressors^14^. The current study reveals that loss of endogenous levels of ARC results in marked increases in CHOP in wild type islets stimulated with ER stress or even, at baseline, in islets obtained from *ob/ob* mice. Taken together with previous work linking CHOP with β-cell apoptosis^20, 21^, the rescue of β-cell death and islet structural abnormalities resulting from loss of ARC indicates that endogenous ARC protects against these phenotypes by suppressing CHOP during type 2 diabetes.

## Methods

### Mice

All mice were back bred >6 generations onto a C57BL/6J background. We previously generated germ line ARC −/− mice^16, 17^. *ob/ob* and CHOP −/− mice were purchased from Jackson Laboratories (Bar Harbor, ME). Male mice of the indicated ages were used. Littermates of respective genotypes were compared. All studies were approved by, and carried out in accordance with, the Institute for Animals Studies at Albert Einstein College of Medicine.

### Metabolic characterization of mice

Blood glucose concentrations were measured in mice fed *ad libitum* or after an overnight fast using OneTouch glucose monitoring system (LifeScan). For glucose tolerance tests (GTT), mice were injected with 2 g glucose/kg body weight after an overnight fast and blood glucose concentrations measured. For glucose-induced insulin secretion (GIIS), plasma insulin and C-peptide concentrations were measured by ELISA (ALPCO Diagnostics) following GTT. For insulin tolerance test (ITT), mice were injected with 5 U insulin/kg body weight after a 6 h fast.

### Immunohistochemistry and quantification

Pancreatic tissue was prepared and stained as previously described^14^. Primary antibodies were ARC (Cayman Chemical), insulin (Abcam), BrdU (Roche), cleaved caspase-3 (Cell Signaling Technology), and α-tubulin (Sigma-Aldrich). Alexa Fluor 488 and 568 (Invitrogen) of corresponding species were used for recognition of primary antibodies. All images were collected with Axio Observer.Z1 microscope (Zeiss). Islet morphology was designated as abnormal if >25% of perimeter was jagged on hematoxylin and eosin-stained sections. All islets (~85–100) in pancreatic section were scored. β-cell area was determined by calculating the number of β-cell nuclei (insulin-positive cells) in an islet divided by the respective islet area (quantified with ImageJ; National Institutes of Health). A minimum of five islets were scored per mouse. For BrdU staining, BrdU 2.2 mg/ml was included in the drinking water for 4 days prior to sacrifice, and tissue sections were treated with 2 M HCl for 10 minutes at room temperature immediately following antigen retrieval. TUNEL staining was performed as previously described^14^. Slides were coverslipped with VECTASHIELD mounting media with DAPI (Vector Laboratories).

### Immunoblotting

Procedures for isolated islets are as previously described^14^. After isolation of islets by collagenase digestion, lysates were digested by rotation in single lysis buffer. Primary antibodies included ARC (Cayman Chemical), GADD153 (CHOP) (Santa Cruz Biotechnology, F-168), and α-tubulin (Sigma-Aldrich). Secondary antibodies included IRDye 800 and 680 (LI-COR). Membranes were scanned using a LI-COR Odyssey and images quantified with ImageJ.

### Statistics

Data are presented as mean ± SEM. For comparisons between two groups, a two-tailed student’s t-test was performed. Multiple comparisons were analyzed using analysis of variance followed by a Tukey post-hoc test. For metabolic measurements, area under the curve was calculated followed by a two-tailed student’s t- test. GraphPad Prism 5 software (La Jolla, CA) was used for calculations. P < 0.05 was deemed significant.

### Data availability

Data generated or analyzed during this study are included in this published article.
